# Supplementary material for: A Humanized Yeast Phenomic Model of Deoxycytidine Kinase to Predict Genetic Buffering of Nucleoside Analog Cytotoxicity
Source: Genes (Basel). 2019 Sep 30;10(10):770. doi: 10.3390/genes10100770 (PMC6826991; doi:10.3390/genes10100770)
Supplement: Supplementary file 1 [file genes-10-00770-s001.zip › Additional_Files/Additional_File7_GOTermHeatmaps.zip!/Additional_File7_GOTermHeatmaps/nucleic_acid_phosphodiester_bond_hydrolysis.pdf]

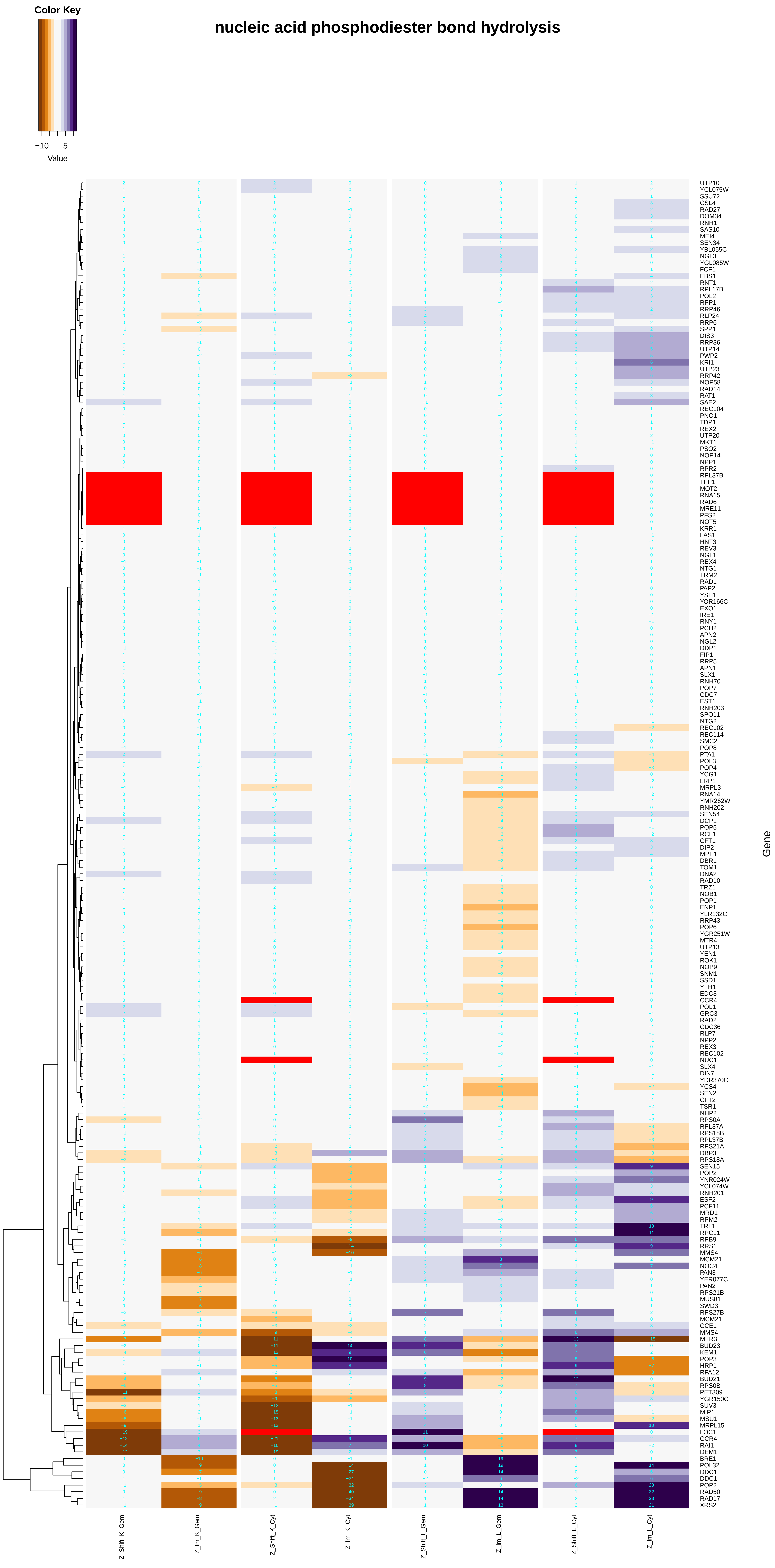
nucleic acid phosphodiester bond hydrolysis
Color Key
−10
5
Value
UTP10
YCL075W
SSU72
CSL4
RAD27
DOM34
RNH1
SAS10
MEI4
SEN34
YBL055C
NGL3
YGL085W
FCF1
EBS1
RNT1
RPL17B
POL2
RPP1
RRP46
RLP24
RRP6
SPP1
DIS3
RRP36
UTP14
PWP2
KRI1
UTP23
RRP42
NOP58
RAD14
RAT1
SAE2
REC104
PNO1
TDP1
REX2
UTP20
MKT1
PSO2
NOP14
NPP1
RPR2
RPL37B
TFP1
MOT2
RNA15
RAD6
MRE11
PFS2
NOT5
KRR1
LAS1
HNT3
REV3
NGL1
REX4
NTG1
TRM2
RAD1
PAP2
YSH1
YOR166C
EXO1
IRE1
RNY1
PCH2
APN2
NGL2
DDP1
FIP1
RRP5
APN1
SLX1
RNH70
POP7
CDC7
EST1
RNH203
SPO11
NTG2
REC102
REC114
SMC2
POP8
PTA1
POL3
POP4
YCG1
LRP1
MRPL3
RNA14
YMR262W
RNH202
SEN54
DCP1
POP5
RCL1
CFT1
DIP2
MPE1
DBR1
TOM1
DNA2
RAD10
TRZ1
NOB1
POP1
ENP1
YLR132C
RRP43
POP6
YGR251W
MTR4
UTP13
YEN1
ROK1
NOP9
SNM1
SSD1
YTH1
EDC3
CCR4
POL1
GRC3
RAD2
CDC36
RLP7
NPP2
REX3
REC102
NUC1
SLX4
DIN7
YDR370C
YCS4
SEN2
CFT2
TSR1
NHP2
RPS0A
RPL37A
RPS18B
RPL37B
RPS21A
DBP3
RPS18A
SEN15
POP2
YNR024W
YCL074W
RNH201
ESF2
PCF11
MRD1
RPM2
TRL1
RPC11
RPB9
RRS1
MMS4
MCM21
NOC4
PAN3
YER077C
PAN2
RPS21B
MUS81
SWD3
RPS27B
MCM21
CCE1
MMS4
MTR3
BUD23
KEM1
POP3
HRP1
RPA12
BUD21
RPS0B
PET309
YGR150C
SUV3
MIP1
MSU1
MRPL15
LOC1
CCR4
RAI1
DEM1
BRE1
POL32
DDC1
DDC1
POP2
RAD50
RAD17
XRS2
Gene
Z_Shift_K_Gem
Z_Im_K_Gem
Z_Shift_K_Cyt
Z_Im_K_Cyt
Z_Shift_L_Gem
Z_Im_L_Gem
Z_Shift_L_Cyt
Z_Im_L_Cyt

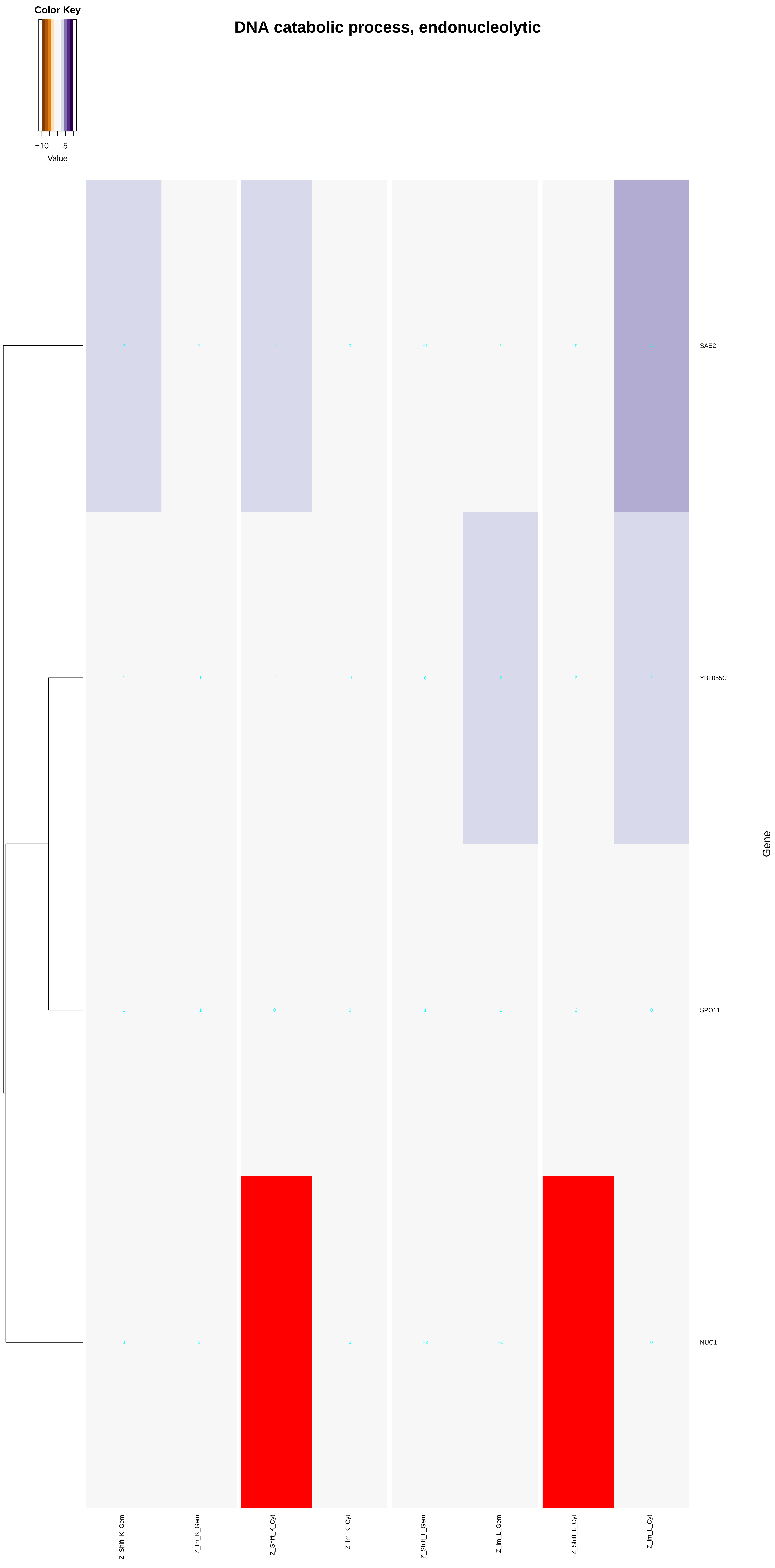
DNA catabolic process, endonucleolytic
Color Key
−10
5
Value
SAE2
YBL055C
SPO11
NUC1
Gene
Z_Shift_K_Gem
Z_Im_K_Gem
Z_Shift_K_Cyt
Z_Im_K_Cyt
Z_Shift_L_Gem
Z_Im_L_Gem
Z_Shift_L_Cyt
Z_Im_L_Cyt

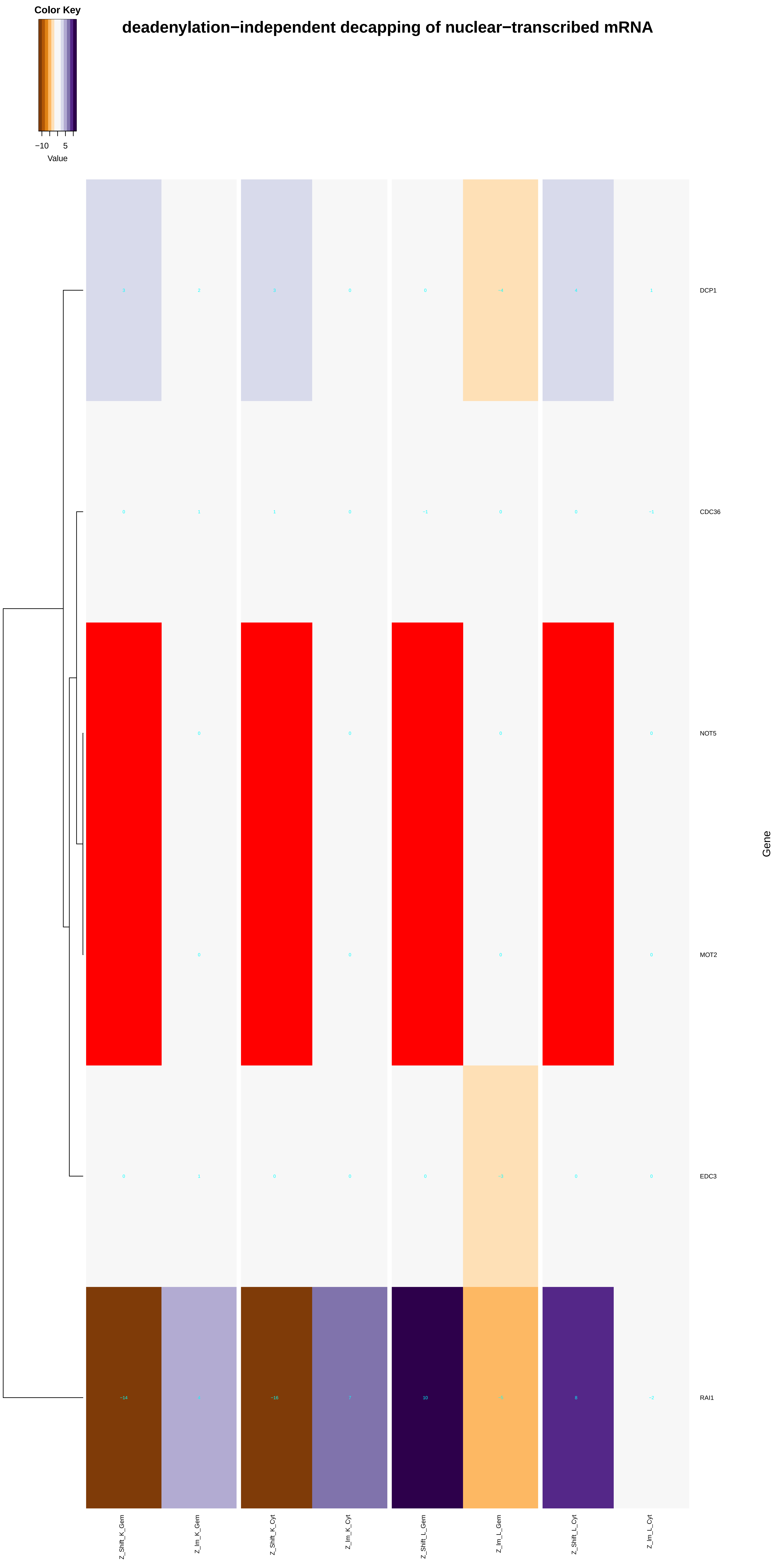
Color Key
−10
5
Value
deadenylation−independent decapping of nuclear−transcribed mRNA
DCP1
CDC36
NOT5
MOT2
EDC3
RAI1
Gene
Z_Shift_K_Gem
Z_Im_K_Gem
Z_Shift_K_Cyt
Z_Im_K_Cyt
Z_Shift_L_Gem
Z_Im_L_Gem
Z_Shift_L_Cyt
Z_Im_L_Cyt

nucleotide−excision repair, DNA incision

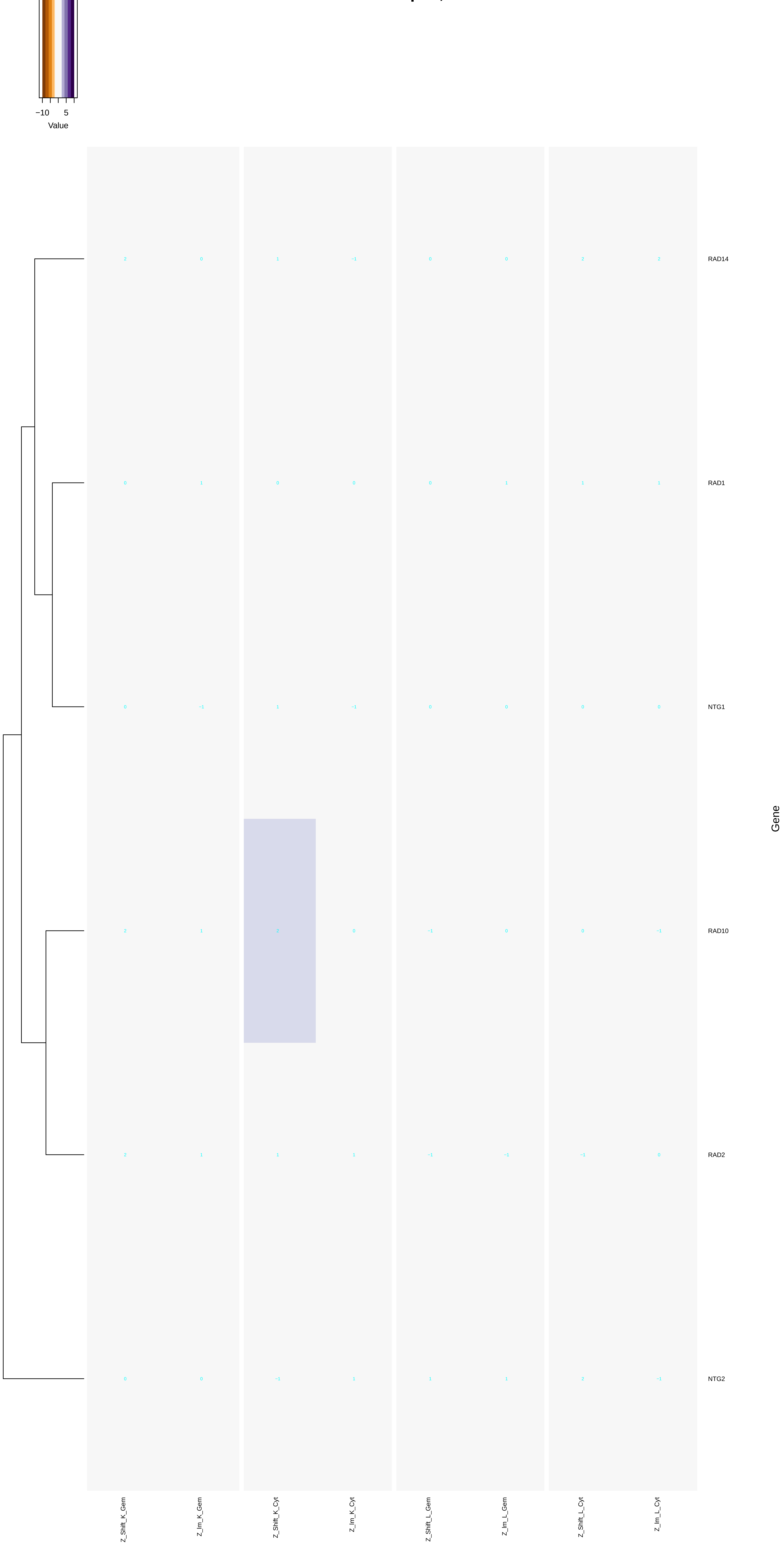

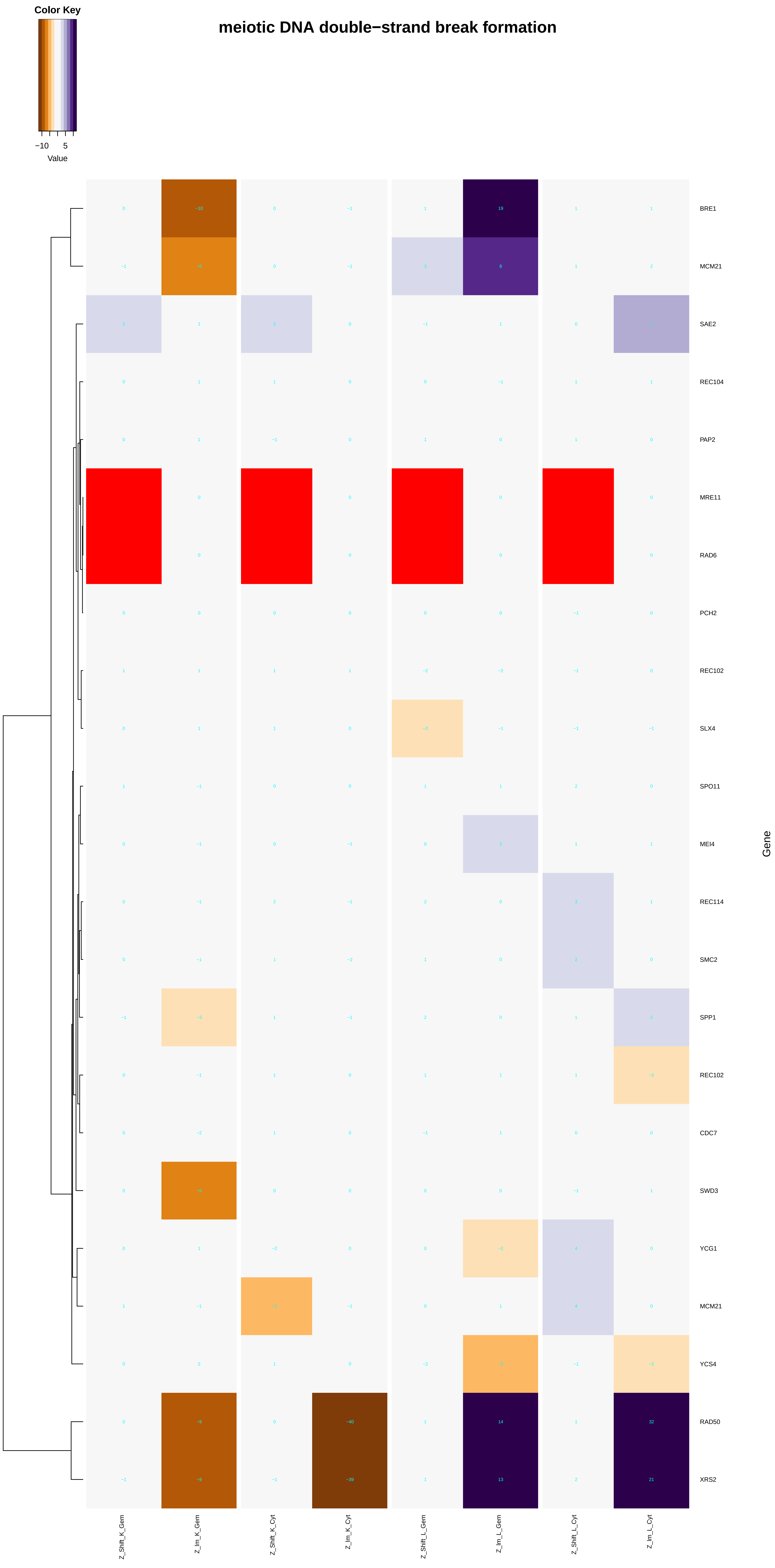
Color Key
−10
5
Value
meiotic DNA double−strand break formation
BRE1
MCM21
SAE2
REC104
PAP2
MRE11
RAD6
PCH2
REC102
SLX4
SPO11
MEI4
REC114
SMC2
SPP1
REC102
CDC7
SWD3
YCG1
MCM21
YCS4
RAD50
XRS2
Gene
Z_Shift_K_Gem
Z_Im_K_Gem
Z_Shift_K_Cyt
Z_Im_K_Cyt
Z_Shift_L_Gem
Z_Im_L_Gem
Z_Shift_L_Cyt
Z_Im_L_Cyt

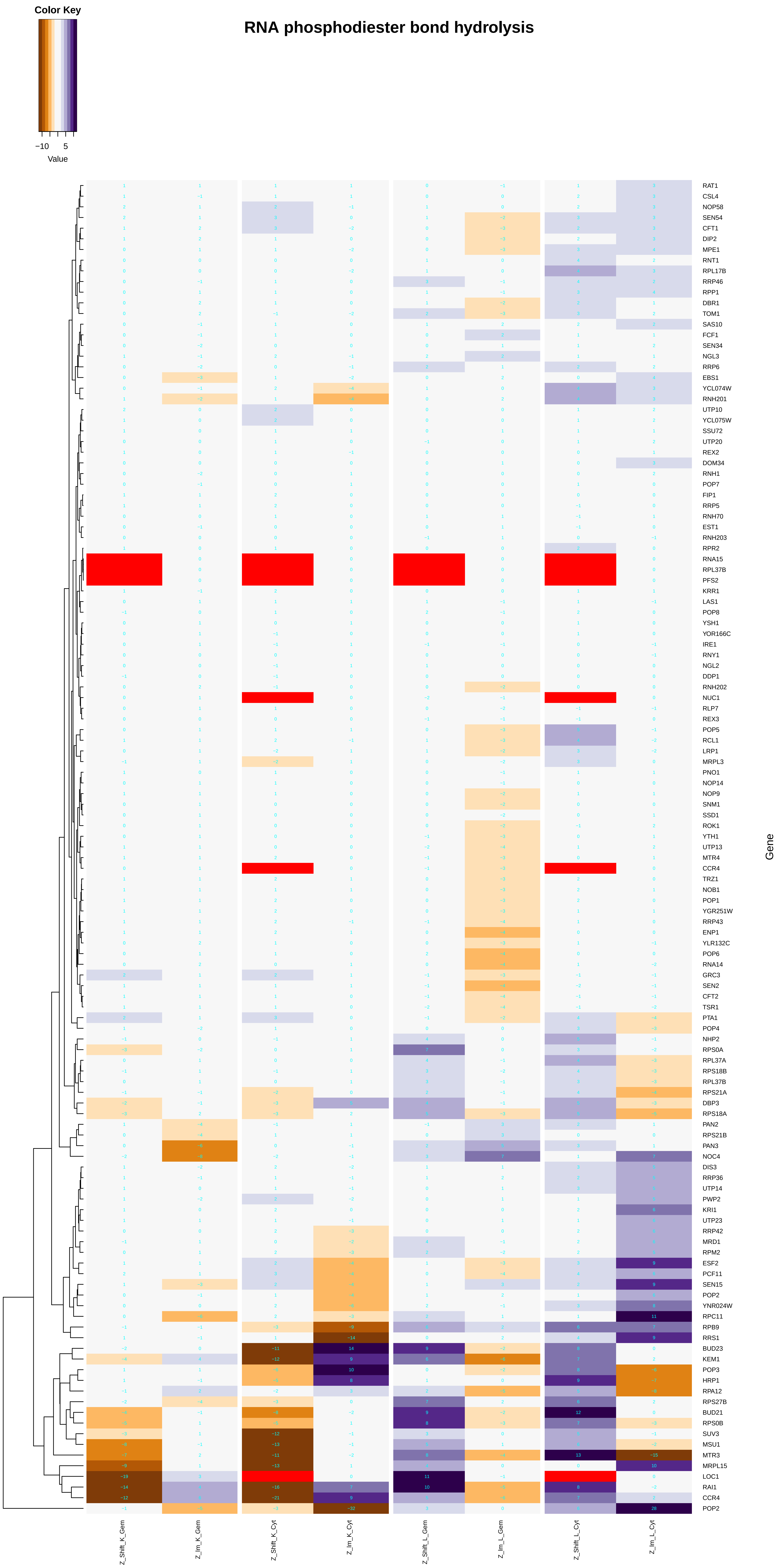
RNA phosphodiester bond hydrolysis
Color Key
−10 5
Value
Gene
RAT1
CSL4
NOP58
SEN54
CFT1
DIP2
MPE1
RNT1
RPL17B
RRP46
RPP1
DBR1
TOM1
SAS10
FCF1
SEN34
NGL3
RRP6
EBS1
YCL074W
RNH201
UTP10
YCL075W
SSU72
UTP20
REX2
DOM34
RNH1
POP7
FIP1
RRP5
RNH70
EST1
RNH203
RPR2
RNA15
RPL37B
PFS2
KRR1
LAS1
POP8
YSH1
YOR166C
IRE1
RNY1
NGL2
DDP1
RNH202
NUC1
RLP7
REX3
POP5
RCL1
LRP1
MRPL3
PNO1
NOP14
NOP9
SNM1
SSD1
ROK1
YTH1
UTP13
MTR4
CCR4
TRZ1
NOB1
POP1
YGR251W
RRP43
ENP1
YLR132C
POP6
RNA14
GRC3
SEN2
CFT2
TSR1
PTA1
POP4
NHP2
RPS0A
RPL37A
RPS18B
RPL37B
RPS21A
DBP3
RPS18A
PAN2
RPS21B
PAN3
NOC4
DIS3
RRP36
UTP14
PWP2
KRI1
UTP23
RRP42
MRD1
RPM2
ESF2
PCF11
SEN15
POP2
YNR024W
RPC11
RPB9
RRS1
BUD23
KEM1
POP3
HRP1
RPA12
RPS27B
BUD21
RPS0B
SUV3
MSU1
MTR3
MRPL15
LOC1
RAI1
CCR4
POP2
Z_Shift_K_Gem
Z_Im_K_Gem
Z_Shift_K_Cyt
Z_Im_K_Cyt
Z_Shift_L_Gem
Z_Im_L_Gem
Z_Shift_L_Cyt
Z_Im_L_Cyt

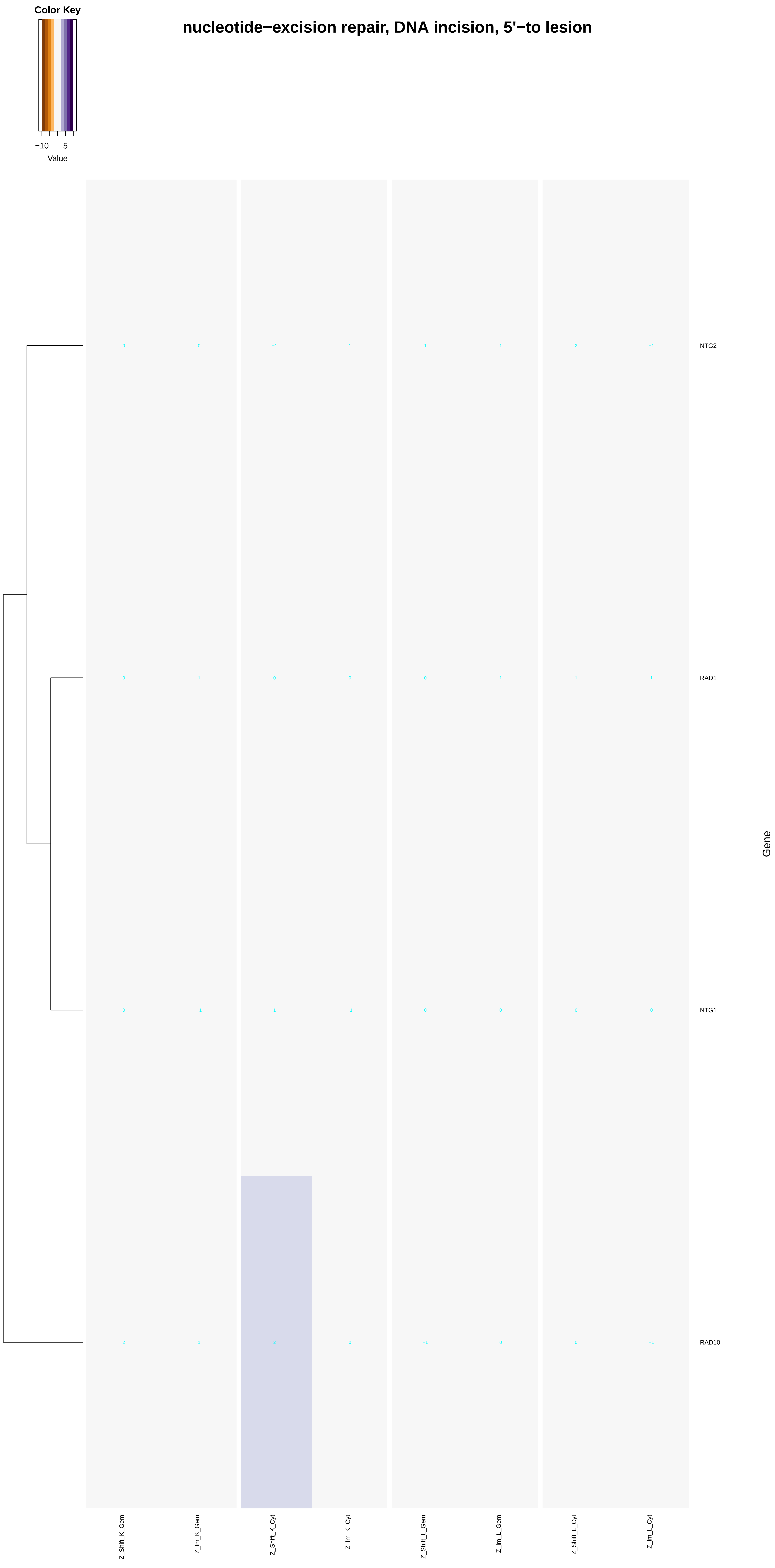
Color Key
−10
5
Value
nucleotide−excision repair, DNA incision, 5'−to lesion
NTG2
RAD1
NTG1
RAD10
Gene
Z_Shift_K_Gem
Z_Im_K_Gem
Z_Shift_K_Cyt
Z_Im_K_Cyt
Z_Shift_L_Gem
Z_Im_L_Gem
Z_Shift_L_Cyt
Z_Im_L_Cyt

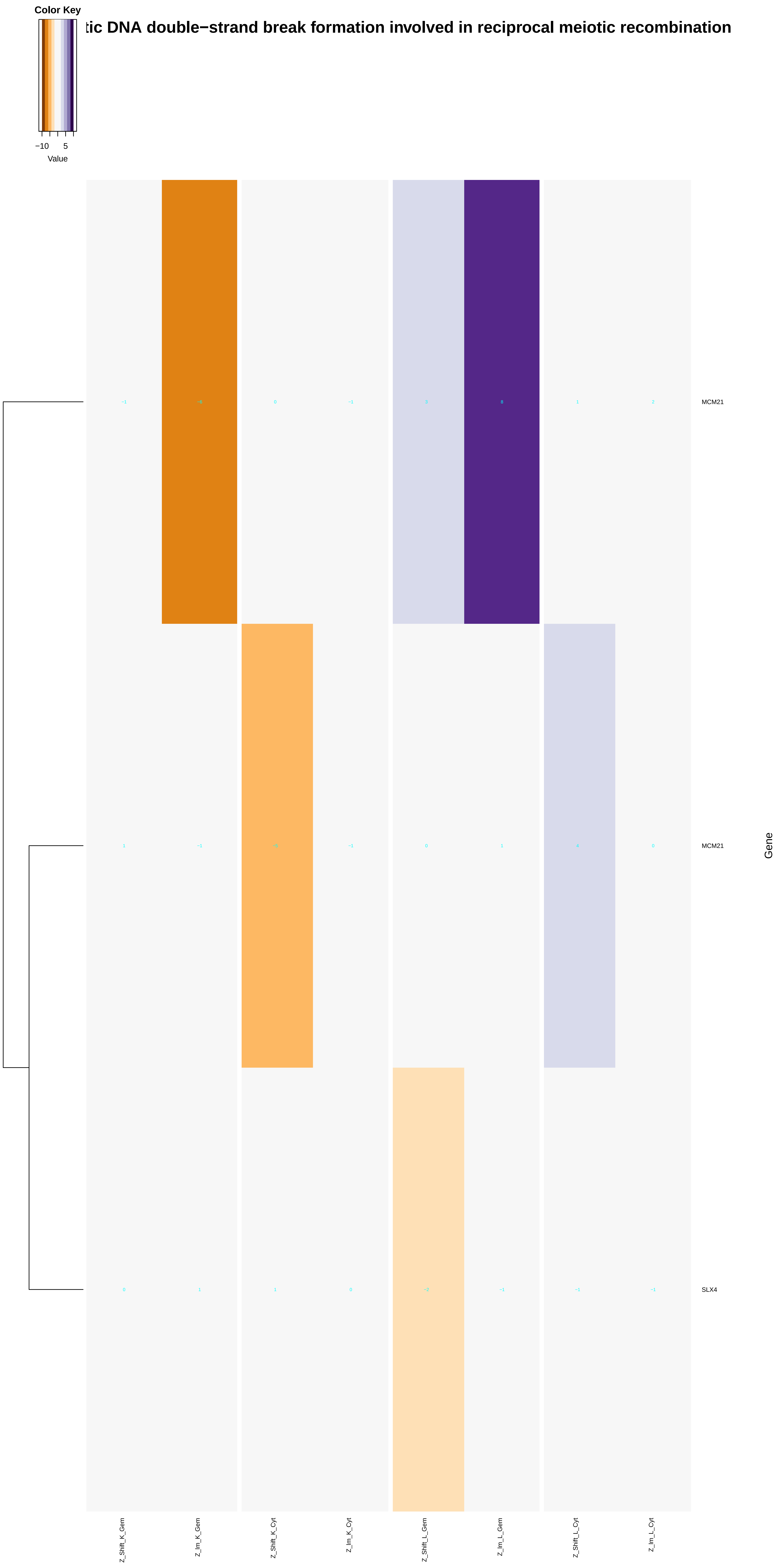
Color Key
−10
5
Value
tic DNA double−strand break formation involved in reciprocal meiotic recombination
−1
−6
0
−1
3
8
1
2
MCM21
1
−1
−5
−1
0
1
4
0
MCM21
0
1
1
0
−2
−1
−1
−1
SLX4
Gene
Z_Shift_K_Gem
Z_Im_K_Gem
Z_Shift_K_Cyt
Z_Im_K_Cyt
Z_Shift_L_Gem
Z_Im_L_Gem
Z_Shift_L_Cyt
Z_Im_L_Cyt

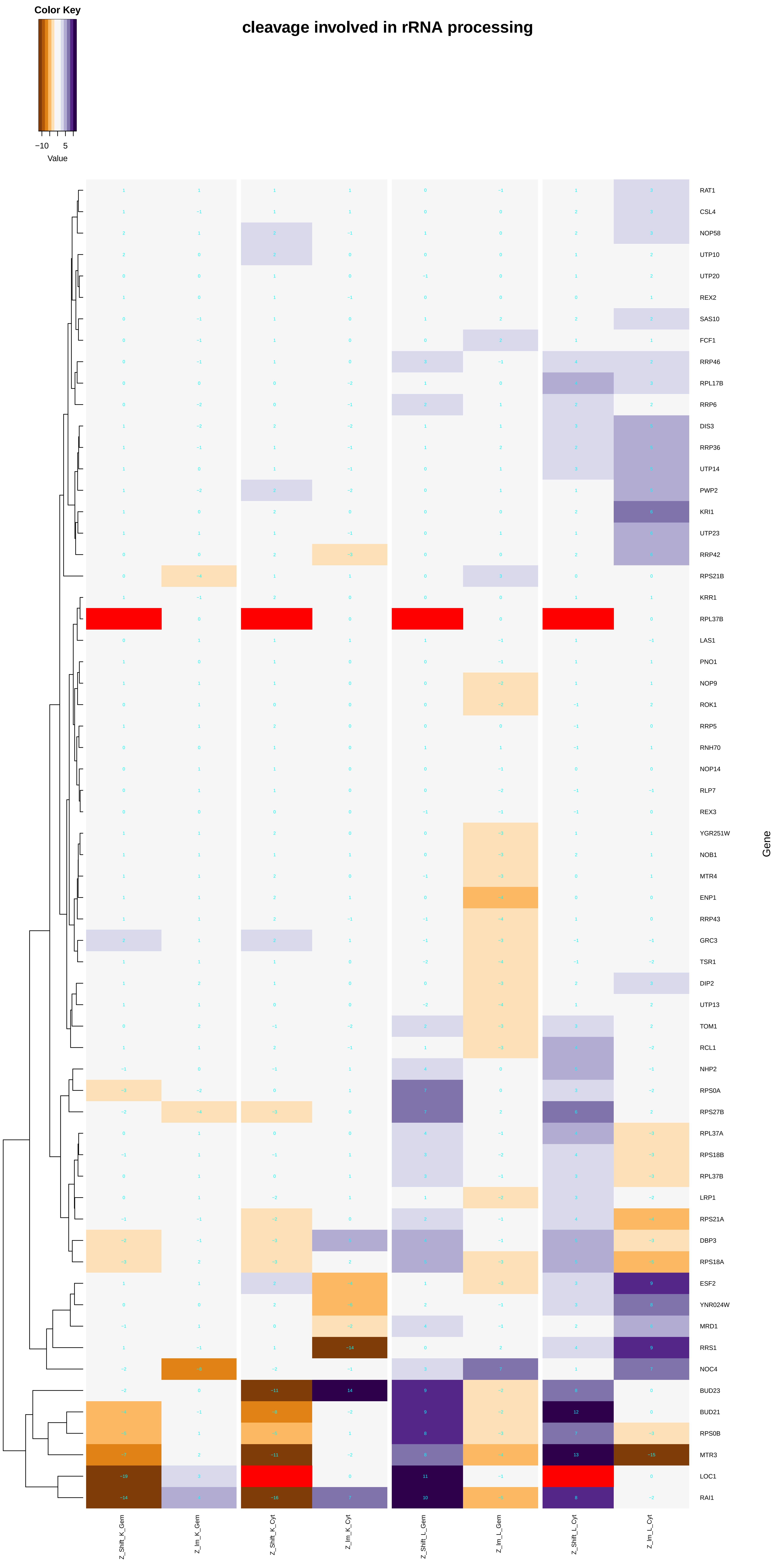
Color Key
−10
5
Value
cleavage involved in rRNA processing
RAT1
CSL4
NOP58
UTP10
UTP20
REX2
SAS10
FCF1
RRP46
RPL17B
RRP6
DIS3
RRP36
UTP14
PWP2
KRI1
UTP23
RRP42
RPS21B
KRR1
RPL37B
LAS1
PNO1
NOP9
ROK1
RRP5
RNH70
NOP14
RLP7
REX3
YGR251W
NOB1
MTR4
ENP1
RRP43
GRC3
TSR1
DIP2
UTP13
TOM1
RCL1
NHP2
RPS0A
RPS27B
RPL37A
RPS18B
RPL37B
LRP1
RPS21A
DBP3
RPS18A
ESF2
YNR024W
MRD1
RRS1
NOC4
BUD23
BUD21
RPS0B
MTR3
LOC1
RAI1
Gene
Z_Shift_K_Gem
Z_Im_K_Gem
Z_Shift_K_Cyt
Z_Im_K_Cyt
Z_Shift_L_Gem
Z_Im_L_Gem
Z_Shift_L_Cyt
Z_Im_L_Cyt

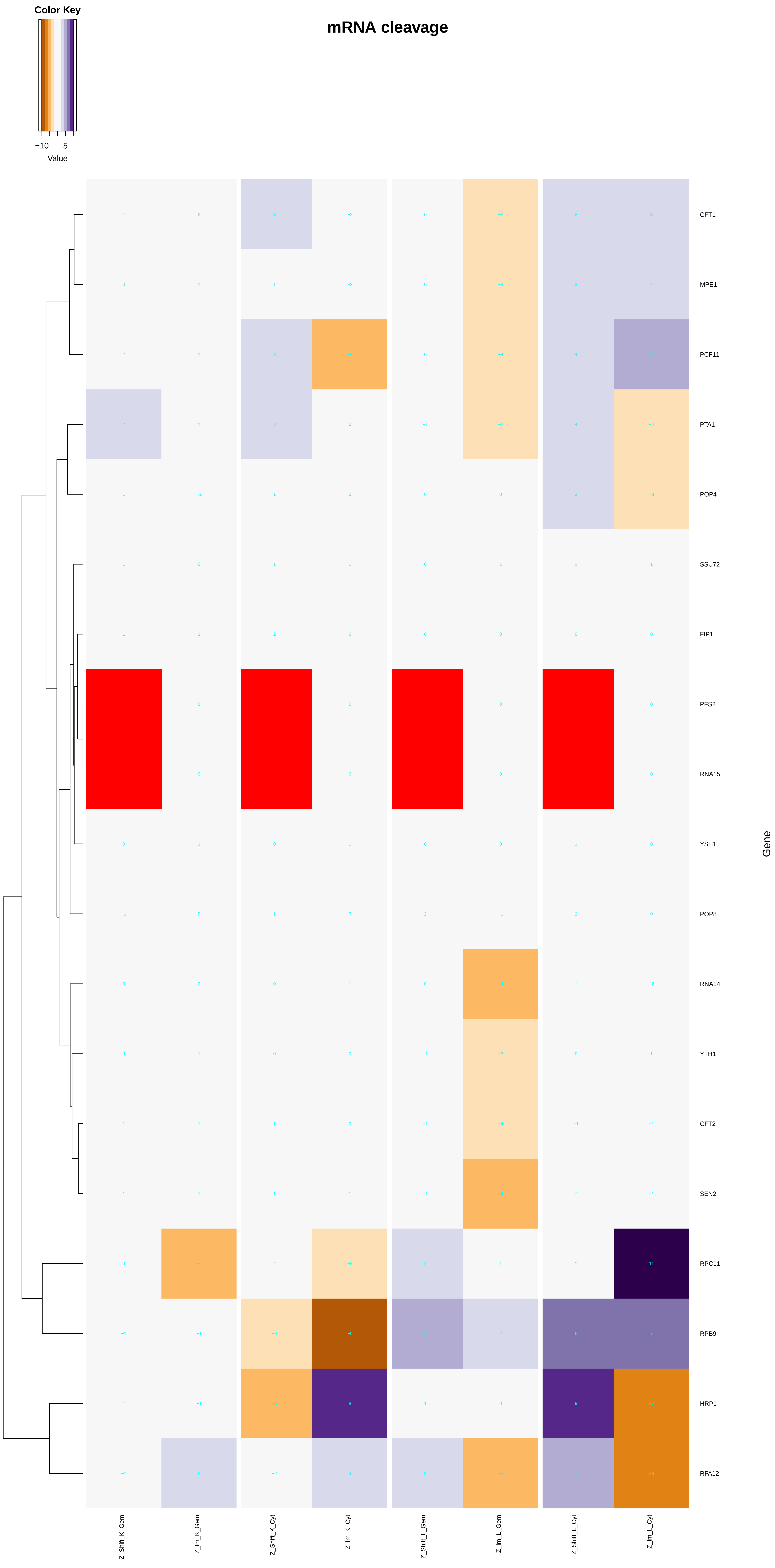
Color Key
−10
5
Value
mRNA cleavage
CFT1
MPE1
PCF11
PTA1
POP4
SSU72
FIP1
PFS2
RNA15
YSH1
POP8
RNA14
YTH1
CFT2
SEN2
RPC11
RPB9
HRP1
RPA12
Gene
Z_Shift_K_Gem
Z_Im_K_Gem
Z_Shift_K_Cyt
Z_Im_K_Cyt
Z_Shift_L_Gem
Z_Im_L_Gem
Z_Shift_L_Cyt
Z_Im_L_Cyt

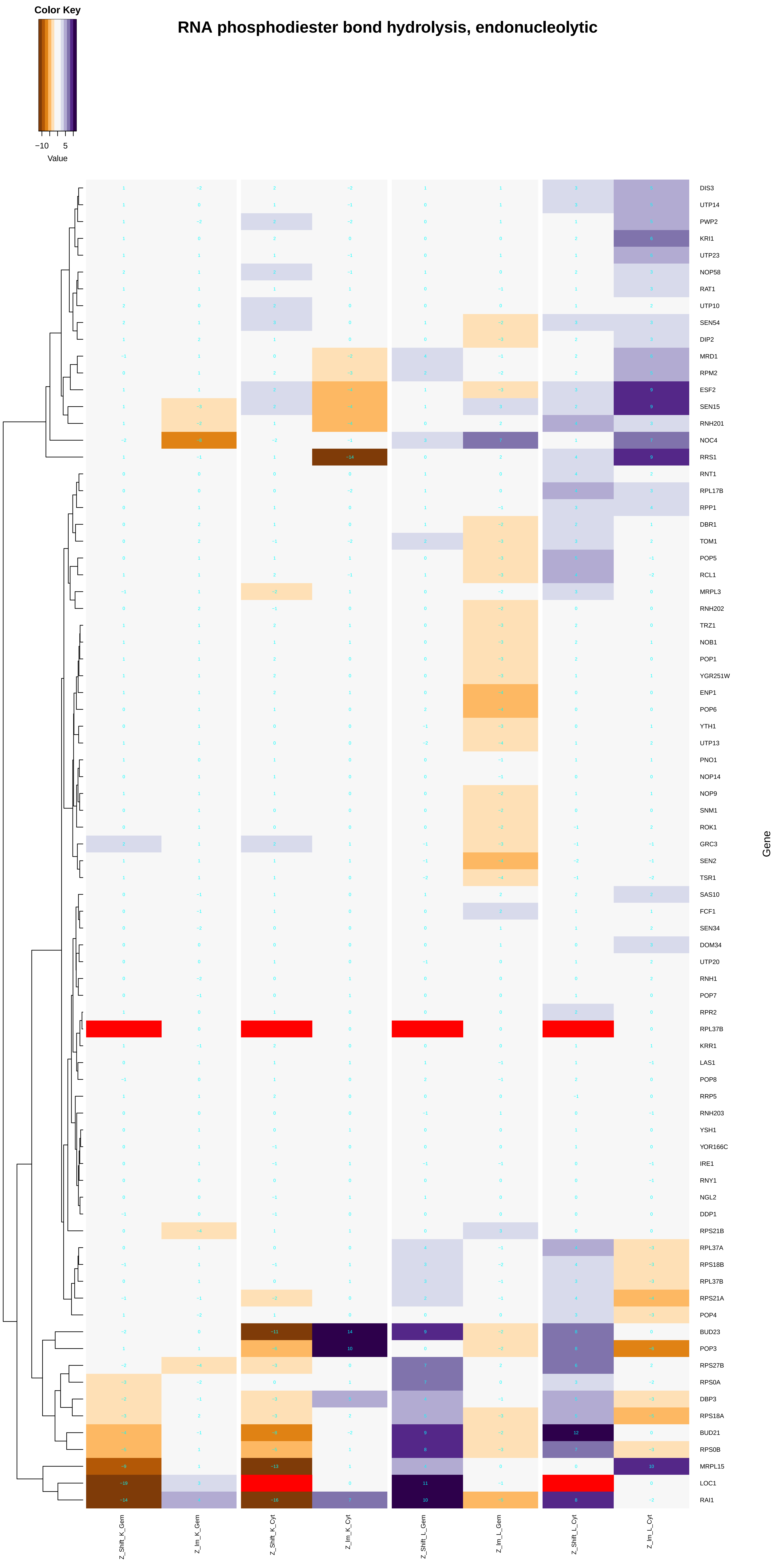
Color Key
−10
5
Value
RNA phosphodiester bond hydrolysis, endonucleolytic
DIS3
UTP14
PWP2
KRI1
UTP23
NOP58
RAT1
UTP10
SEN54
DIP2
MRD1
RPM2
ESF2
SEN15
RNH201
NOC4
RRS1
RNT1
RPL17B
RPP1
DBR1
TOM1
POP5
RCL1
MRPL3
RNH202
TRZ1
NOB1
POP1
YGR251W
ENP1
POP6
YTH1
UTP13
PNO1
NOP14
NOP9
SNM1
ROK1
GRC3
SEN2
TSR1
SAS10
FCF1
SEN34
DOM34
UTP20
RNH1
POP7
RPR2
RPL37B
KRR1
LAS1
POP8
RRP5
RNH203
YSH1
YOR166C
IRE1
RNY1
NGL2
DDP1
RPS21B
RPL37A
RPS18B
RPL37B
RPS21A
POP4
BUD23
POP3
RPS27B
RPS0A
DBP3
RPS18A
BUD21
RPS0B
MRPL15
LOC1
RAI1
Gene
Z_Shift_K_Gem
Z_Im_K_Gem
Z_Shift_K_Cyt
Z_Im_K_Cyt
Z_Shift_L_Gem
Z_Im_L_Gem
Z_Shift_L_Cyt
Z_Im_L_Cyt

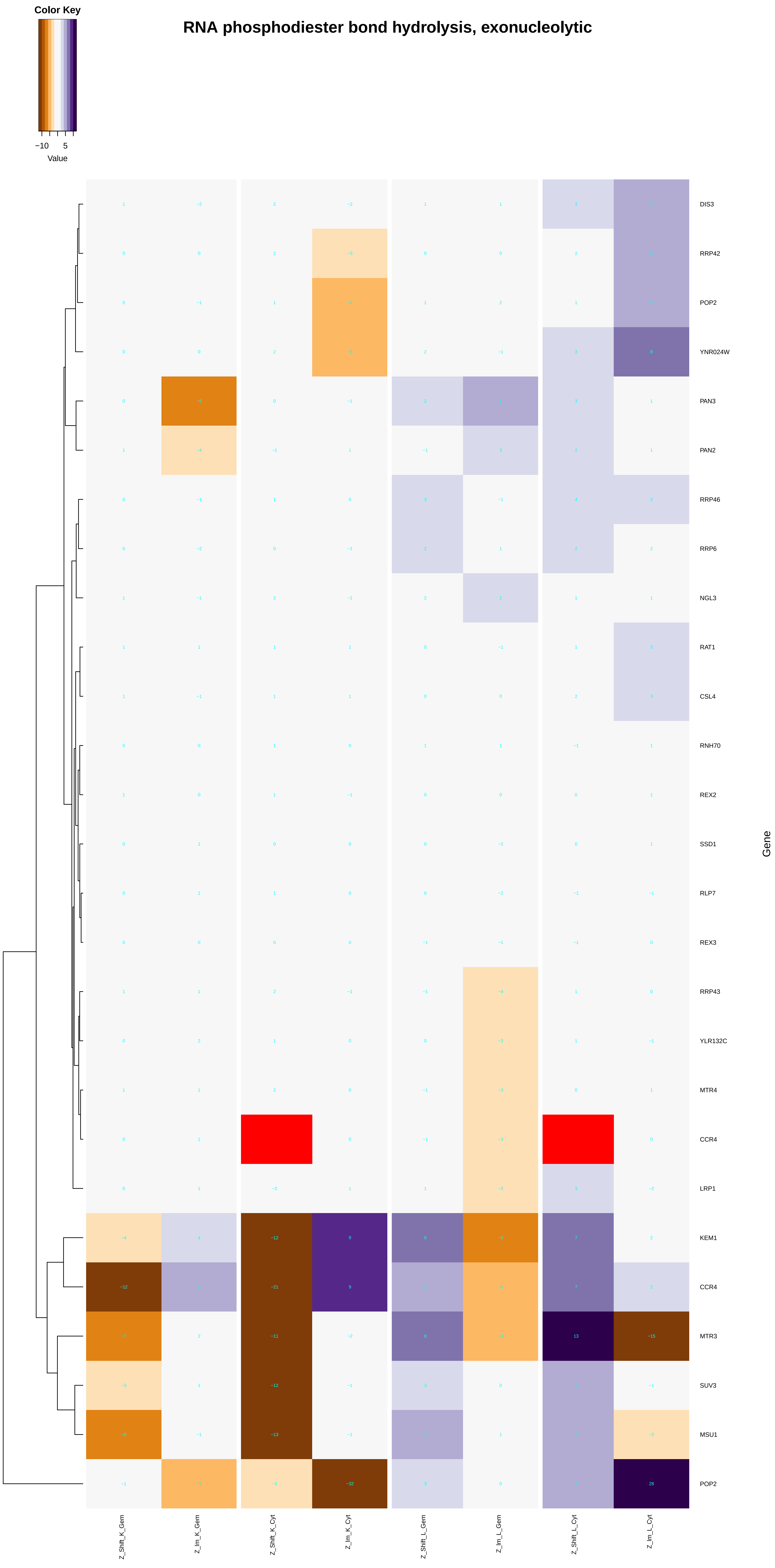
RNA phosphodiester bond hydrolysis, exonucleolytic
Color Key
−10
5
Value
DIS3
RRP42
POP2
YNR024W
PAN3
PAN2
RRP46
RRP6
NGL3
RAT1
CSL4
RNH70
REX2
SSD1
RLP7
REX3
RRP43
YLR132C
MTR4
CCR4
LRP1
KEM1
CCR4
MTR3
SUV3
MSU1
POP2
Gene
Z_Shift_K_Gem
Z_Im_K_Gem
Z_Shift_K_Cyt
Z_Im_K_Cyt
Z_Shift_L_Gem
Z_Im_L_Gem
Z_Shift_L_Cyt
Z_Im_L_Cyt

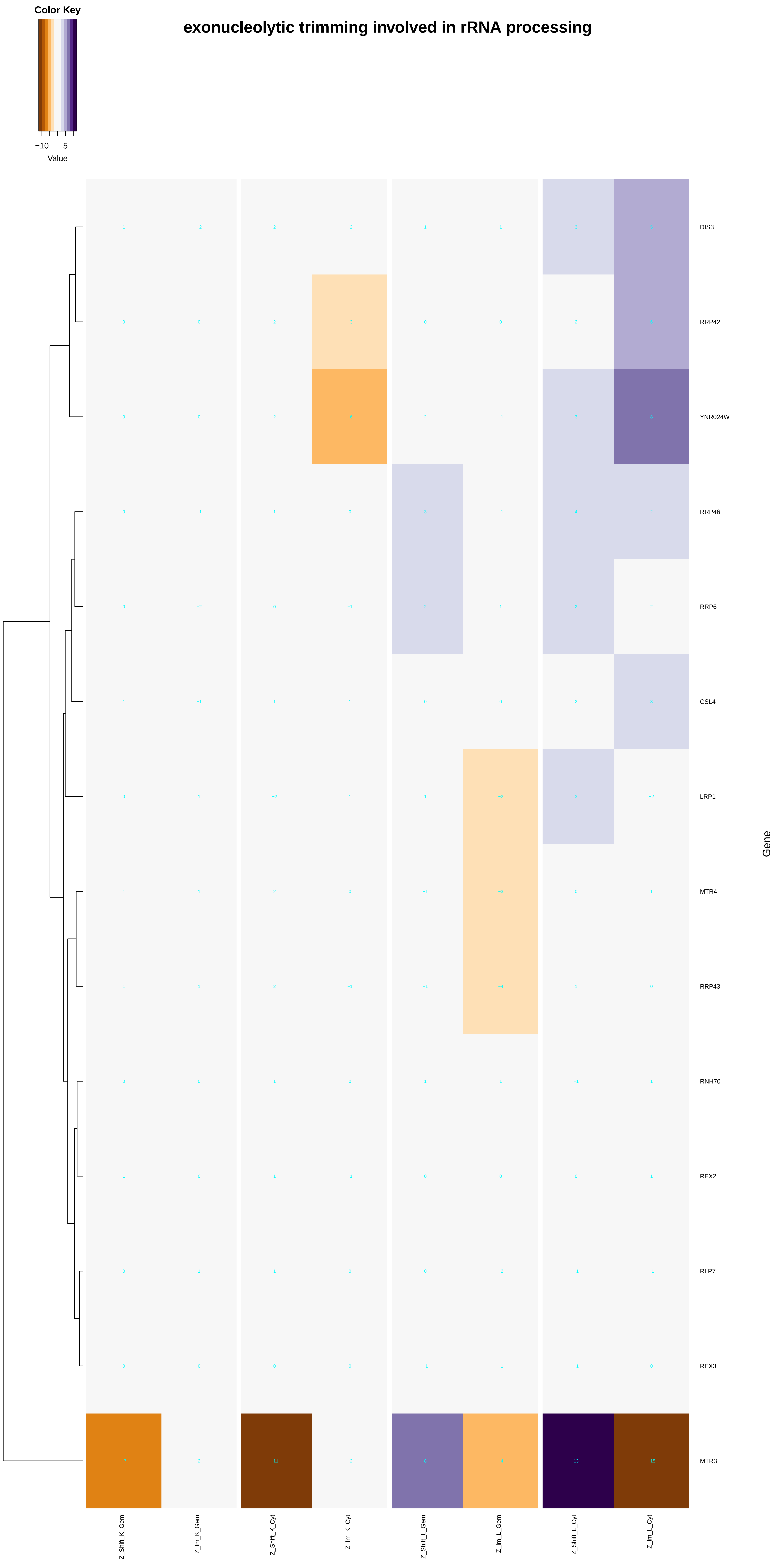

Color Key
−10
5
Value
exonucleolytic trimming involved in rRNA processing
DIS3
RRP42
YNR024W
RRP46
RRP6
CSL4
LRP1
MTR4
RRP43
RNH70
REX2
RLP7
REX3
MTR3
Gene
Z_Shift_K_Gem
Z_Im_K_Gem
Z_Shift_K_Cyt
Z_Im_K_Cyt
Z_Shift_L_Gem
Z_Im_L_Gem
Z_Shift_L_Cyt
Z_Im_L_Cyt

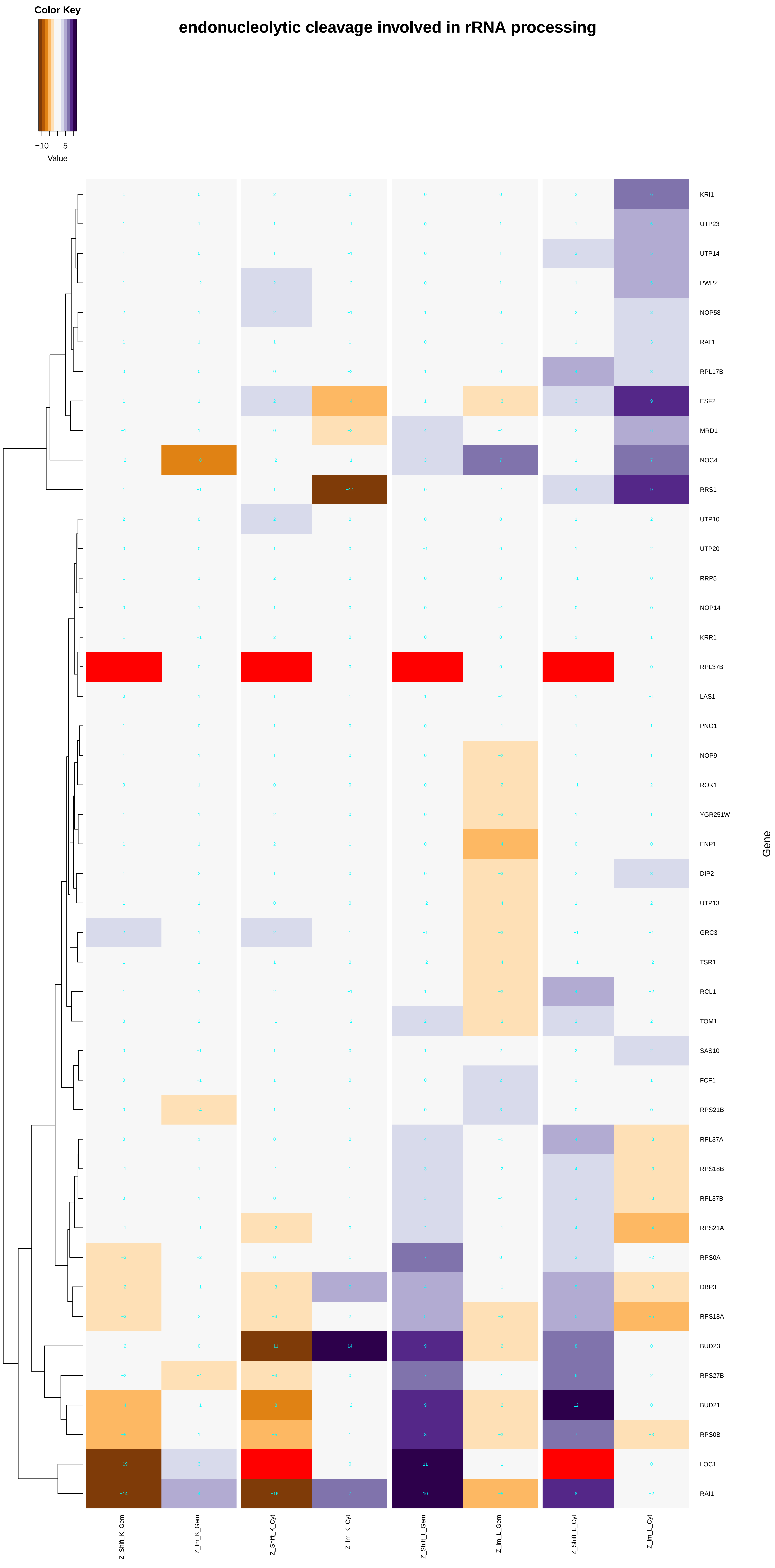
Color Key
−10 5
Value
endonucleolytic cleavage involved in rRNA processing
KRI1
UTP23
UTP14
PWP2
NOP58
RAT1
RPL17B
ESF2
MRD1
NOC4
RRS1
UTP10
UTP20
RRP5
NOP14
KRR1
RPL37B
LAS1
PNO1
NOP9
ROK1
YGR251W
ENP1
DIP2
UTP13
GRC3
TSR1
RCL1
TOM1
SAS10
FCF1
RPS21B
RPL37A
RPS18B
RPL37B
RPS21A
RPS0A
DBP3
RPS18A
BUD23
RPS27B
BUD21
RPS0B
LOC1
RAI1
Gene
Z_Shift_K_Gem
Z_Im_K_Gem
Z_Shift_K_Cyt
Z_Im_K_Cyt
Z_Shift_L_Gem
Z_Im_L_Gem
Z_Shift_L_Cyt
Z_Im_L_Cyt

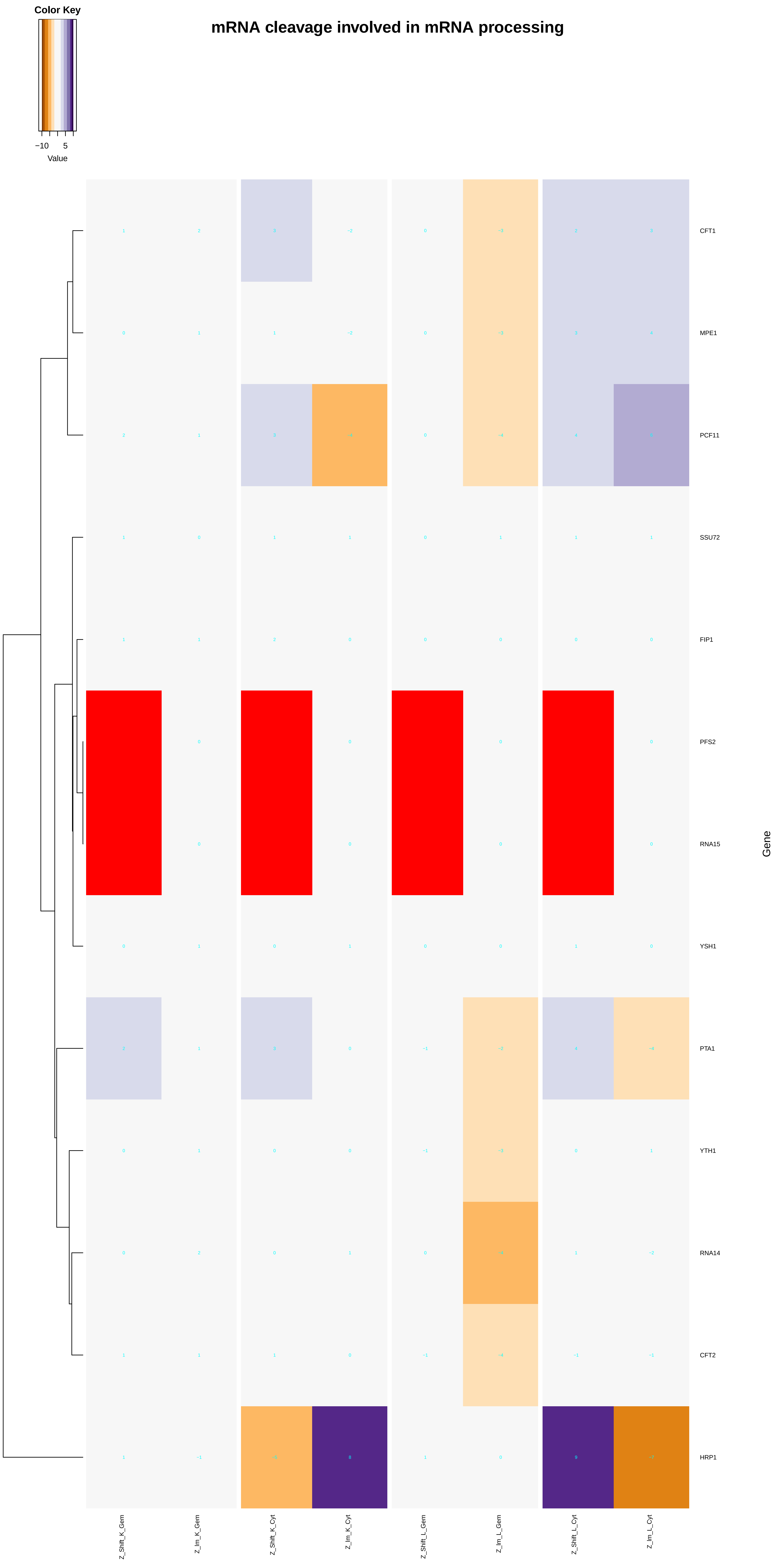
mRNA cleavage involved in mRNA processing
Color Key
−10
5
Value
CFT1
MPE1
PCF11
SSU72
FIP1
PFS2
RNA15
YSH1
PTA1
YTH1
RNA14
CFT2
HRP1
Gene
Z_Shift_K_Gem
Z_Im_K_Gem
Z_Shift_K_Cyt
Z_Im_K_Cyt
Z_Shift_L_Gem
Z_Im_L_Gem
Z_Shift_L_Cyt
Z_Im_L_Cyt

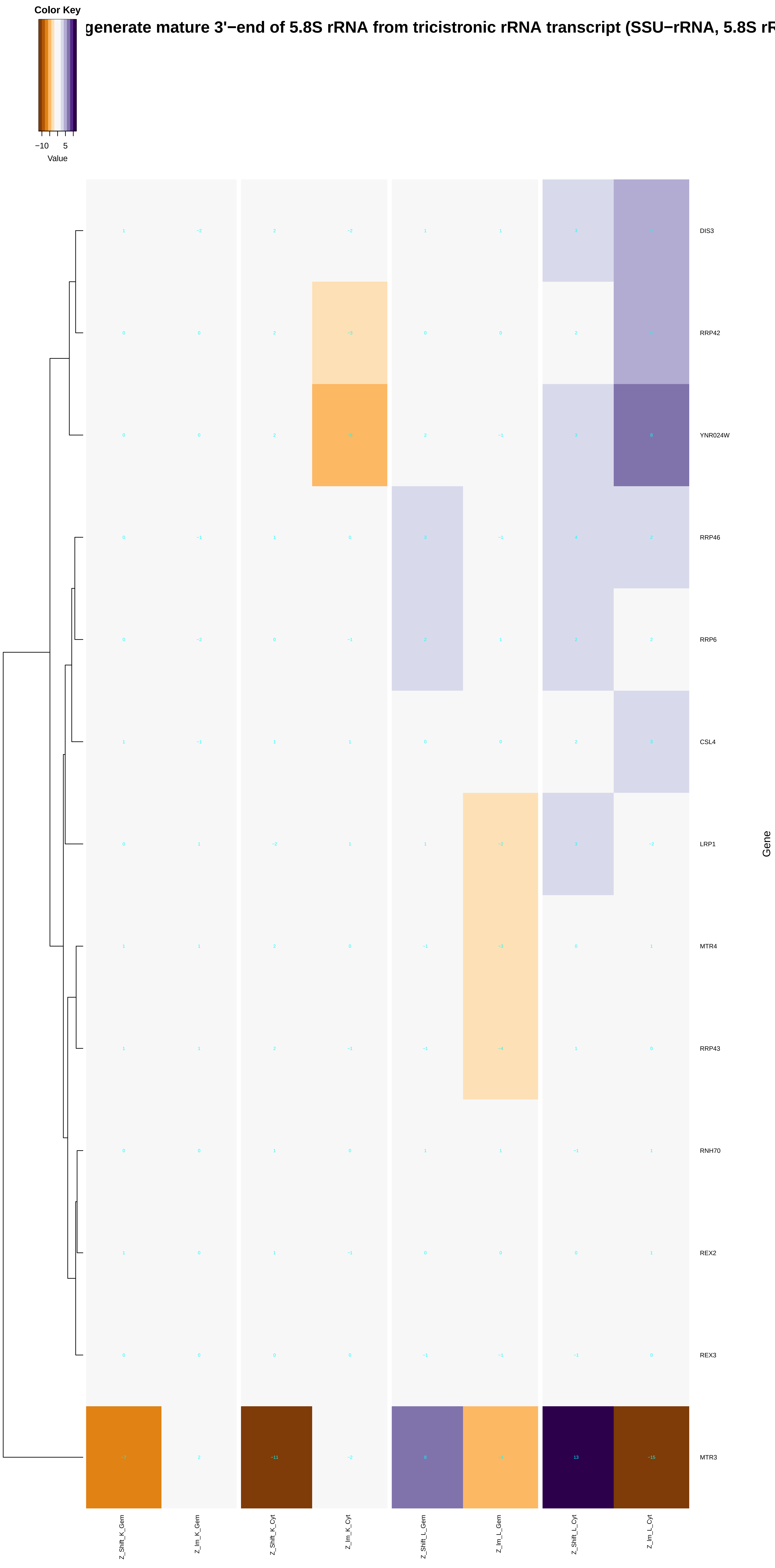
Color Key
−10
5
Value
generate mature 3'−end of 5.8S rRNA from tricistronic rRNA transcript (SSU−rRNA, 5.8S rR
DIS3
RRP42
YNR024W
RRP46
RRP6
CSL4
LRP1
MTR4
RRP43
RNH70
REX2
REX3
MTR3
Gene
Z_Shift_K_Gem
Z_lm_K_Gem
Z_Shift_K_Cyt
Z_lm_K_Cyt
Z_Shift_L_Gem
Z_lm_L_Gem
Z_Shift_L_Cyt
Z_lm_L_Cyt

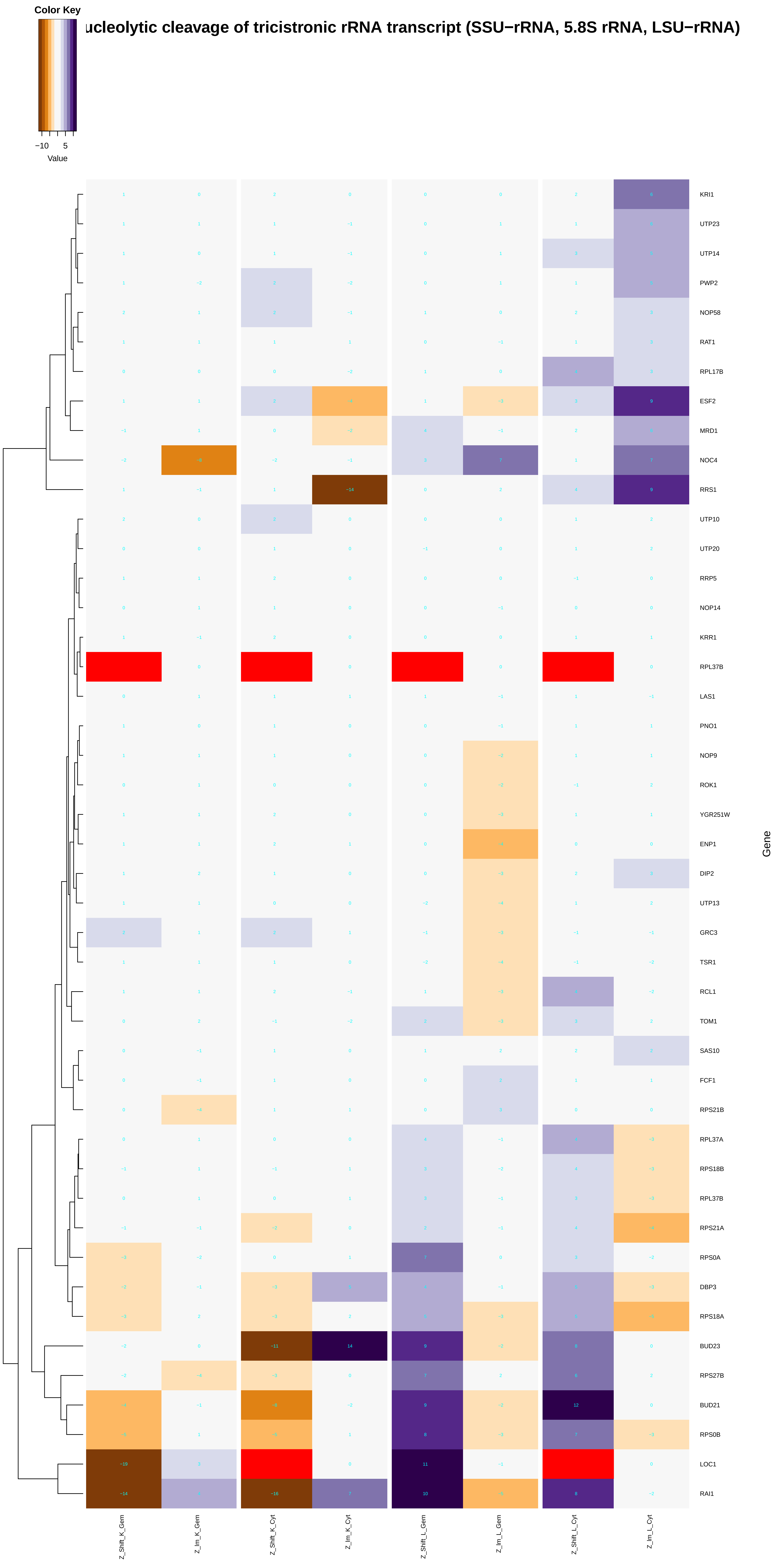

Color Key
−10 5
Value
ucleolytic cleavage of tricistronic rRNA transcript (SSU−rRNA, 5.8S rRNA, LSU−rRNA)
KRI1
UTP23
UTP14
PWP2
NOP58
RAT1
RPL17B
ESF2
MRD1
NOC4
RRS1
UTP10
UTP20
RRP5
NOP14
KRR1
RPL37B
LAS1
PNO1
NOP9
ROK1
YGR251W
ENP1
DIP2
UTP13
GRC3
TSR1
RCL1
TOM1
SAS10
FCF1
RPS21B
RPL37A
RPS18B
RPL37B
RPS21A
RPS0A
DBP3
RPS18A
BUD23
RPS27B
BUD21
RPS0B
LOC1
RAI1
Gene
Z_Shift_K_Gem
Z_Im_K_Gem
Z_Shift_K_Cyt
Z_Im_K_Cyt
Z_Shift_L_Gem
Z_Im_L_Gem
Z_Shift_L_Cyt
Z_Im_L_Cyt

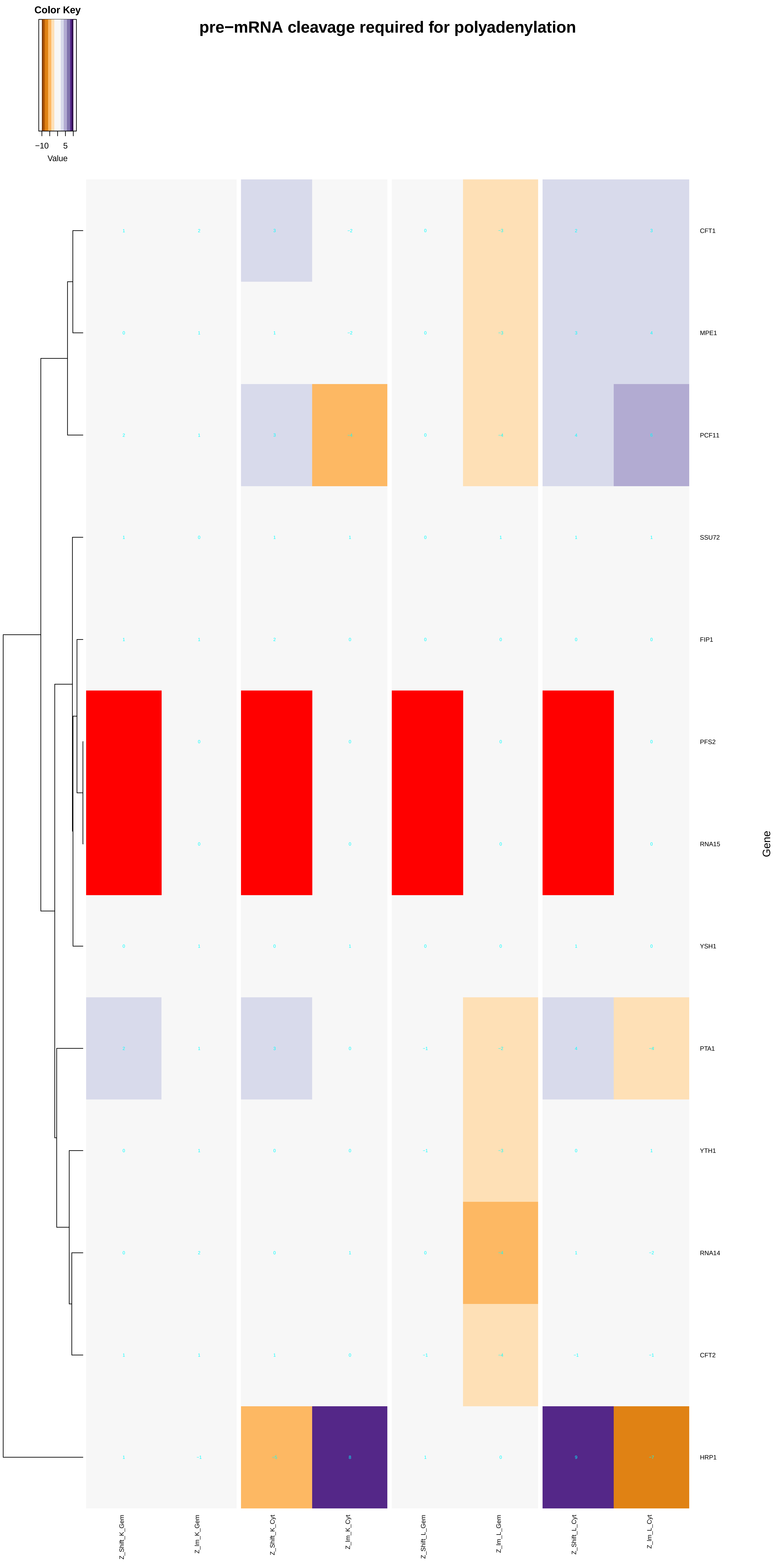
pre−mRNA cleavage required for polyadenylation
Color Key
−10
5
Value
CFT1
MPE1
PCF11
SSU72
FIP1
PFS2
RNA15
YSH1
PTA1
YTH1
RNA14
CFT2
HRP1
Gene
Z_Shift_K_Gem
Z_Im_K_Gem
Z_Shift_K_Cyt
Z_Im_K_Cyt
Z_Shift_L_Gem
Z_Im_L_Gem
Z_Shift_L_Cyt
Z_Im_L_Cyt

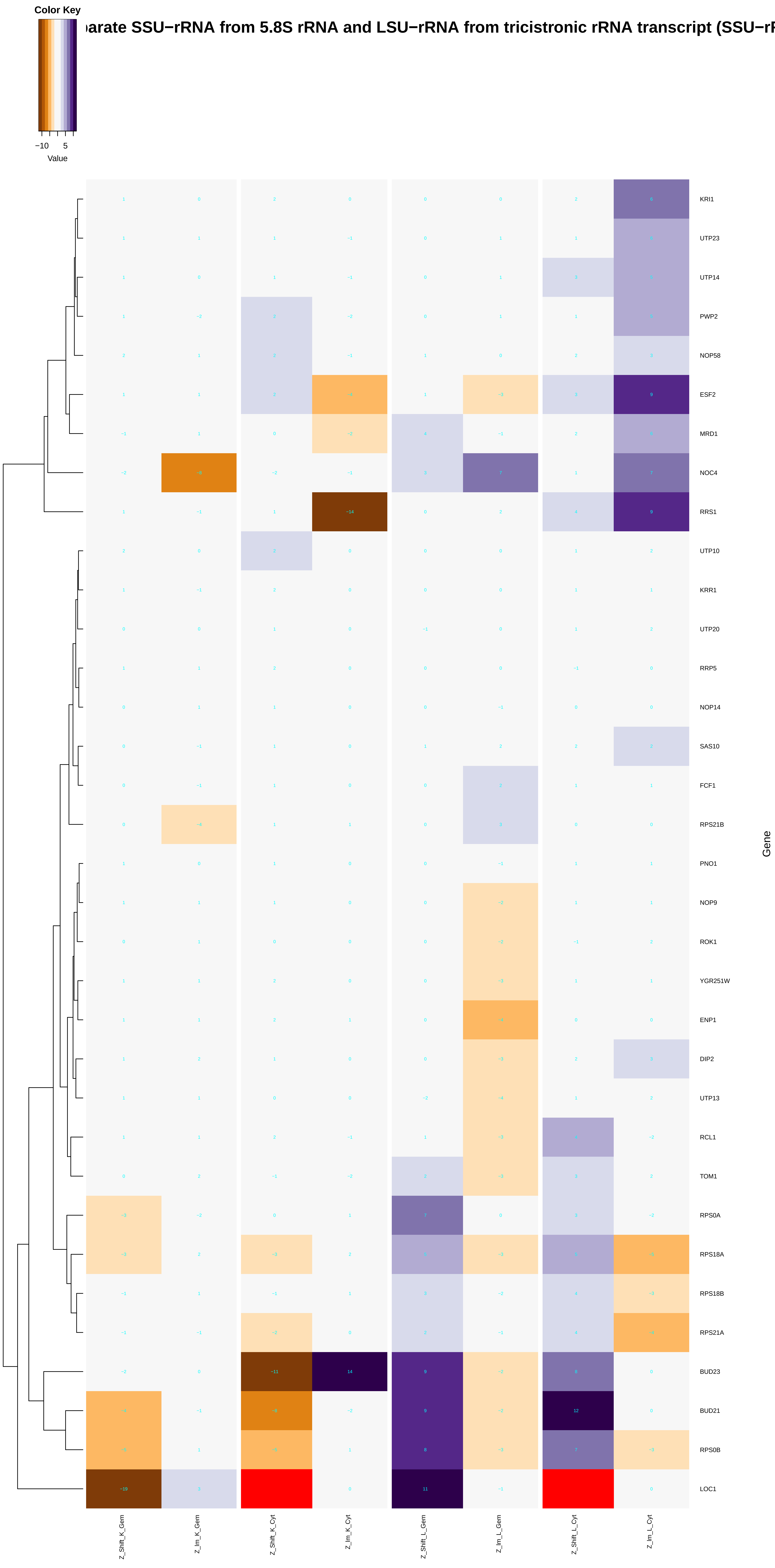

arate SSU−rRNA from 5.8S rRNA and LSU−rRNA from tricistronic rRNA transcript (SSU−rF
Color Key
−10
5
Value
KRI1
UTP23
UTP14
PWP2
NOP58
ESF2
MRD1
NOC4
RRS1
UTP10
KRR1
UTP20
RRP5
NOP14
SAS10
FCF1
RPS21B
PNO1
NOP9
ROK1
YGR251W
ENP1
DIP2
UTP13
RCL1
TOM1
RPS0A
RPS18A
RPS18B
RPS21A
BUD23
BUD21
RPS0B
LOC1
Gene
Z_Shift_K_Gem
Z_Im_K_Gem
Z_Shift_K_Cyt
Z_Im_K_Cyt
Z_Shift_L_Gem
Z_Im_L_Gem
Z_Shift_L_Cyt
Z_Im_L_Cyt

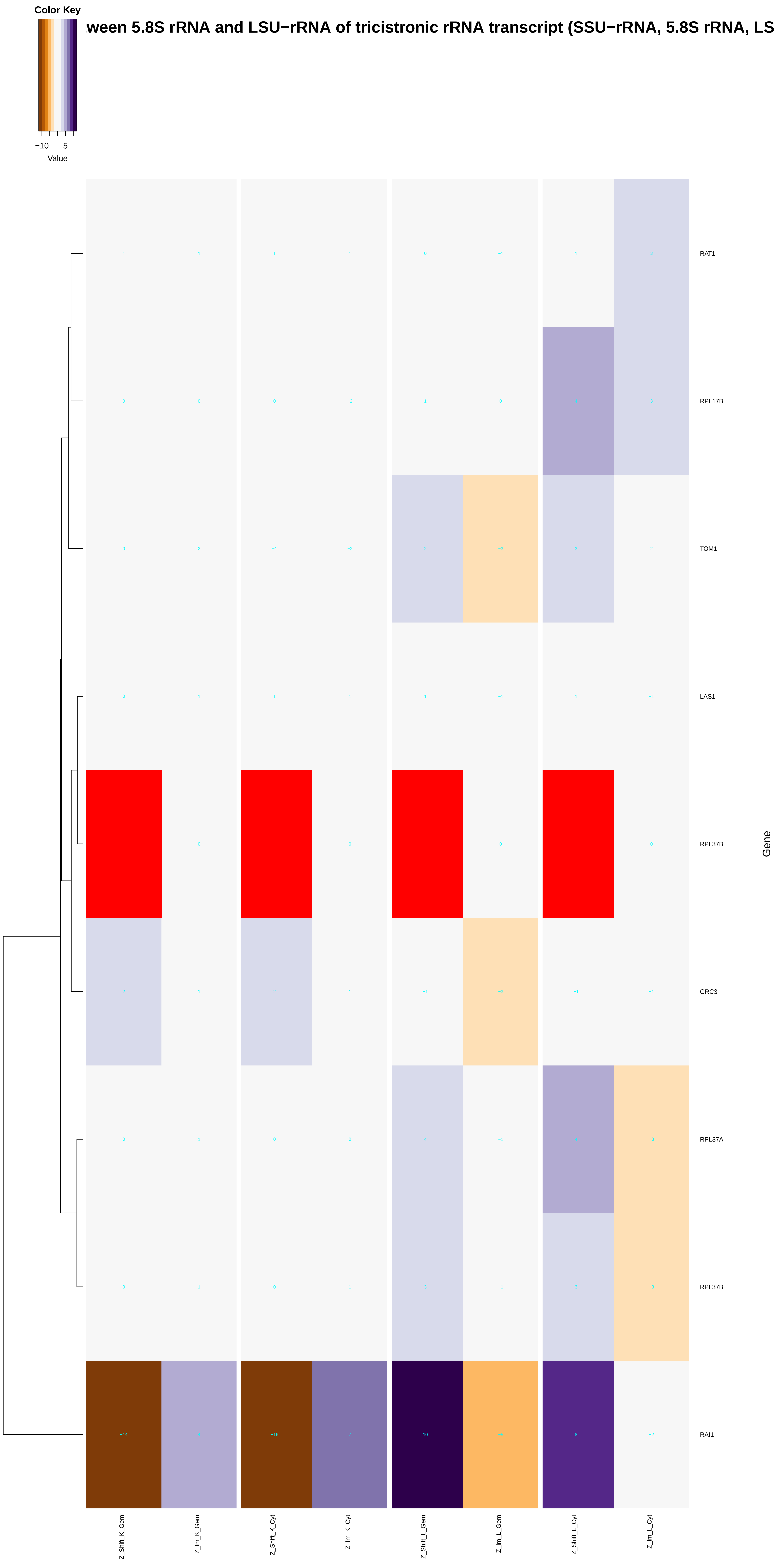
ween 5.8S rRNA and LSU−rRNA of tricistronic rRNA transcript (SSU−rRNA, 5.8S rRNA, LS
Color Key
−10
5
Value
RAT1
RPL17B
TOM1
LAS1
RPL37B
GRC3
RPL37A
RPL37B
RAI1
Gene
Z_Shift_K_Gem
Z_Im_K_Gem
Z_Shift_K_Cyt
Z_Im_K_Cyt
Z_Shift_L_Gem
Z_Im_L_Gem
Z_Shift_L_Cyt
Z_Im_L_Cyt

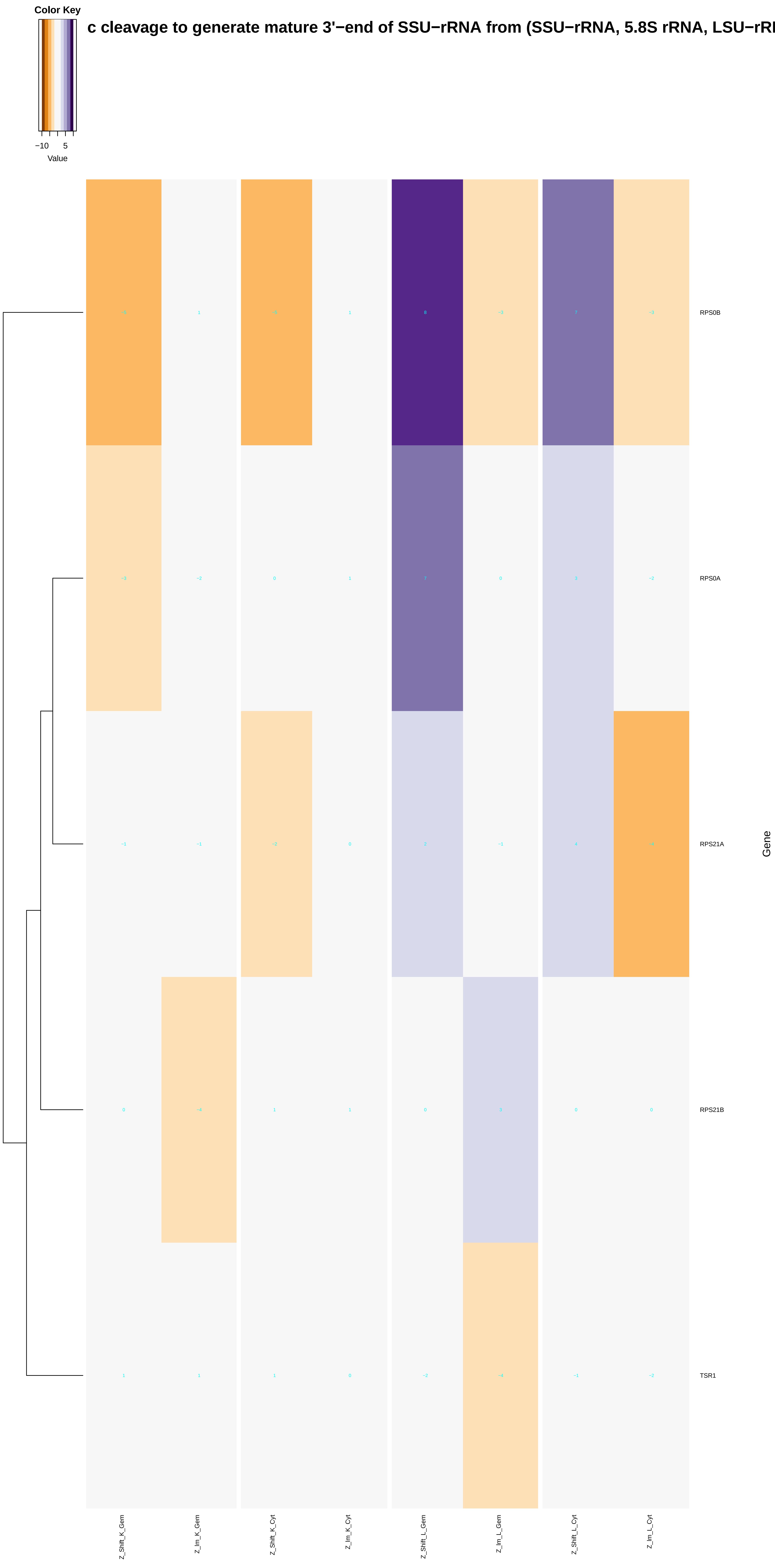
Color Key
−10
5
Value
c cleavage to generate mature 3'−end of SSU−rRNA from (SSU−rRNA, 5.8S rRNA, LSU−rR
RPS0B
RPS0A
RPS21A
RPS21B
TSR1
Gene
Z_Shift_K_Gem
Z_Im_K_Gem
Z_Shift_K_Cyt
Z_Im_K_Cyt
Z_Shift_L_Gem
Z_Im_L_Gem
Z_Shift_L_Cyt
Z_Im_L_Cyt

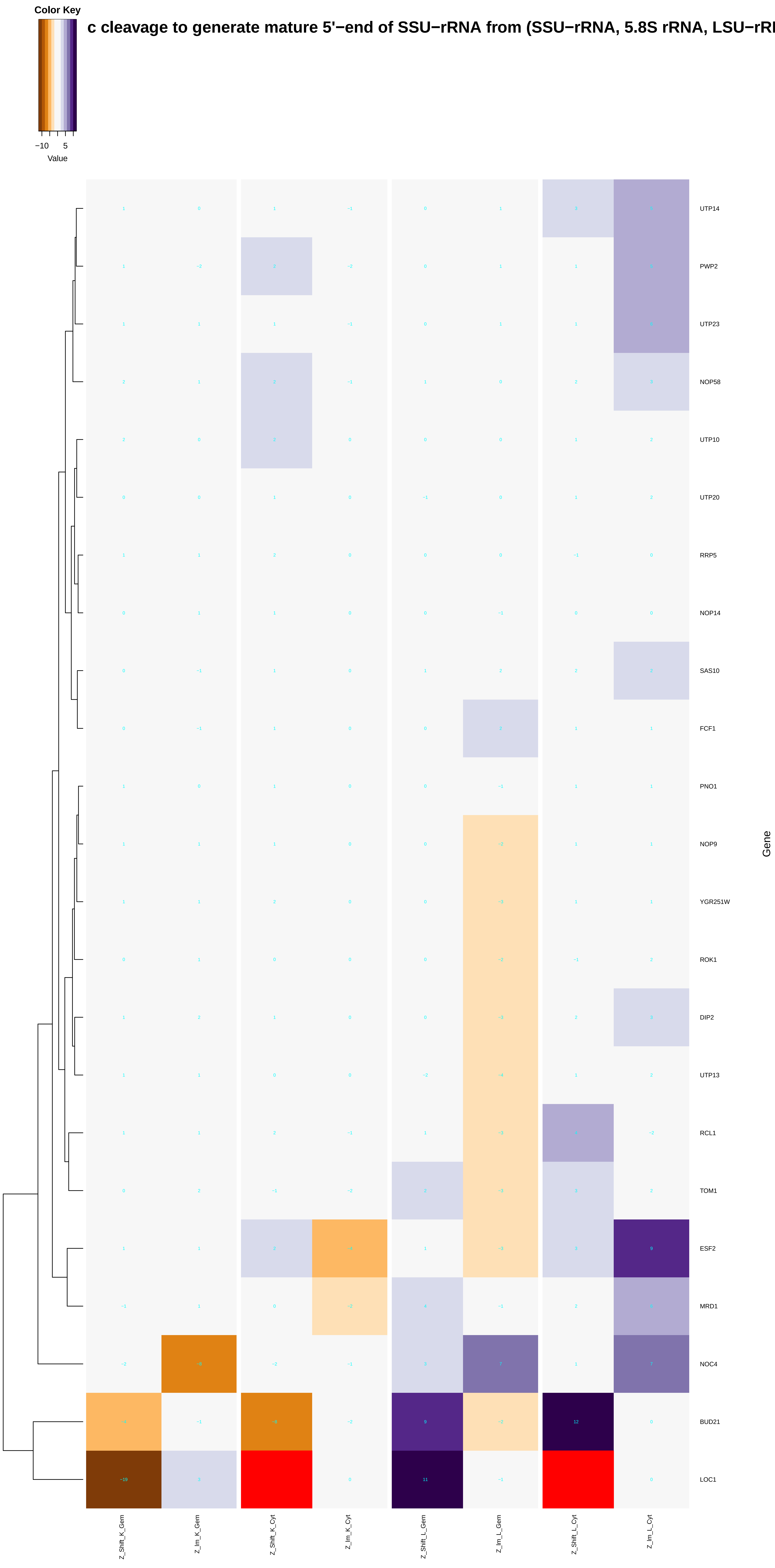

Color Key
−10
5
Value
c cleavage to generate mature 5'−end of SSU−rRNA from (SSU−rRNA, 5.8S rRNA, LSU−rRI
UTP14
PWP2
UTP23
NOP58
UTP10
UTP20
RRP5
NOP14
SAS10
FCF1
PNO1
NOP9
YGR251W
ROK1
DIP2
UTP13
RCL1
TOM1
ESF2
MRD1
NOC4
BUD21
LOC1
Gene
Z_Shift_K_Gem
Z_Im_K_Gem
Z_Shift_K_Cyt
Z_Im_K_Cyt
Z_Shift_L_Gem
Z_Im_L_Gem
Z_Shift_L_Cyt
Z_Im_L_Cyt

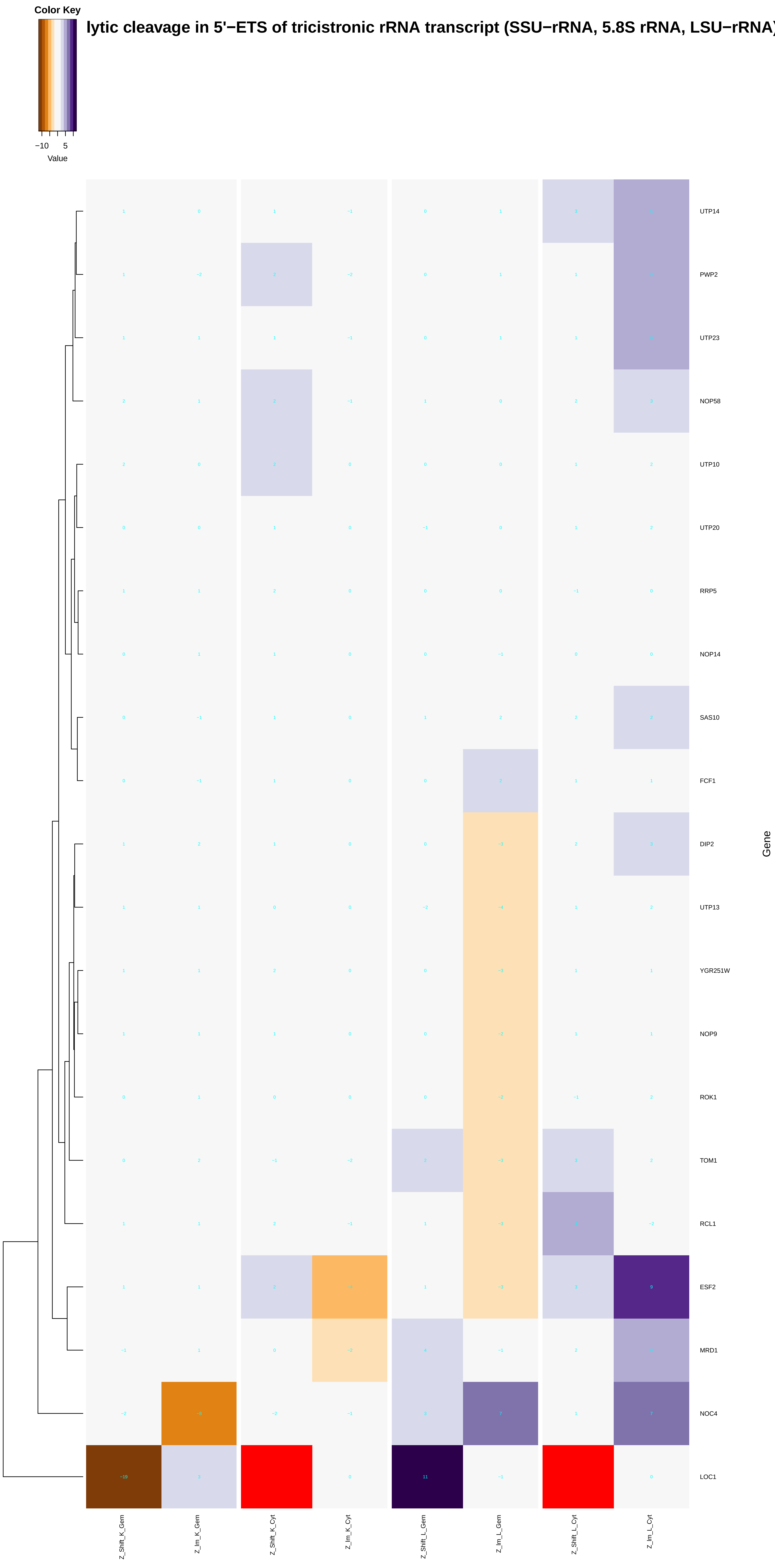
Color Key
−10 5
Value
lytic cleavage in 5'−ETS of tricistronic rRNA transcript (SSU−rRNA, 5.8S rRNA, LSU−rRNA)
UTP14
PWP2
UTP23
NOP58
UTP10
UTP20
RRP5
NOP14
SAS10
FCF1
DIP2
UTP13
YGR251W
NOP9
ROK1
TOM1
RCL1
ESF2
MRD1
NOC4
LOC1
Gene
Z_Shift_K_Gem
Z_lm_K_Gem
Z_Shift_K_Cyt
Z_lm_K_Cyt
Z_Shift_L_Gem
Z_lm_L_Gem
Z_Shift_L_Cyt
Z_lm_L_Cyt
